# Solid-State Dispersions of Platinum in the SnO_2_ and Fe_2_O_3_ Nanomaterials

**DOI:** 10.3390/nano11123349

**Published:** 2021-12-10

**Authors:** Edi Radin, Goran Štefanić, Goran Dražić, Ivan Marić, Tanja Jurkin, Anđela Pustak, Nikola Baran, Matea Raić, Marijan Gotić

**Affiliations:** 1 Laboratory for Molecular Physics and Synthesis of New Materials, Ruđer Bošković Institute, Bijenička c. 54, 10000 Zagreb, Croatia; Edi.Radin@irb.hr (E.R.); Goran.Stefanic@irb.hr (G.Š.); nikola.baran@irb.hr (N.B.); matea.raic@irb.hr (M.R.); 2 National Institute of Chemistry, Hajdrihova 19, SI-1001 Ljubljana, Slovenia; 3 Radiation Chemistry and Dosimetry Laboratory, Ruđer Bošković Institute, Bijenička c. 54, 10000 Zagreb, Croatia; imaric@irb.hr (I.M.); tjurkin@irb.hr (T.J.); Andjela.Pustak@irb.hr (A.P.)

**Keywords:** platinum, hematite, cassiterite, ball-milling, mechanochemical, Fe_2_O_3_, SnO_2_, dispersion, XPS, STEM

## Abstract

The dispersion of platinum (Pt) on metal oxide supports is important for catalytic and gas sensing applications. In this work, we used mechanochemical dispersion and compatible Fe(II) acetate, Sn(II) acetate and Pt(II) acetylacetonate powders to better disperse Pt in Fe_2_O_3_ and SnO_2_. The dispersion of platinum in SnO_2_ is significantly different from the dispersion of Pt over Fe_2_O_3_. Electron microscopy has shown that the elements Sn, O and Pt are homogeneously dispersed in α-SnO_2_ (cassiterite), indicating the formation of a (Pt,Sn)O_2_ solid solution. In contrast, platinum is dispersed in α-Fe_2_O_3_ (hematite) mainly in the form of isolated Pt nanoparticles despite the oxidative conditions during annealing. The size of the dispersed Pt nanoparticles over α-Fe_2_O_3_ can be controlled by changing the experimental conditions and is set to 2.2, 1.2 and 0.8 nm. The rather different Pt dispersion in α-SnO_2_ and α-Fe_2_O_3_ is due to the fact that Pt^4+^ can be stabilized in the α-SnO_2_ structure by replacing Sn^4+^ with Pt^4+^ in the crystal lattice, while the substitution of Fe^3+^ with Pt^4+^ is unfavorable and Pt^4+^ is mainly expelled from the lattice at the surface of α-Fe_2_O_3_ to form isolated platinum nanoparticles.

## 1. Introduction

The nanomaterials based on noble metal nanoparticles and semiconducting transition metal oxides have found applications in electrochemistry, photochemistry, biosensing, catalysis and gas sensing. In particular, the Pt nanoparticles dispersed on the non-expensive SnO_2_ or Fe_2_O_3_ supports have been used in various catalytic reactions or in gas sensing applications. For example, Chen et al. [1] hydrothermally synthesized a Pt/Fe_2_O_3_ nanocomposite catalyst with facet and defect structure for the catalytic oxidation of formaldehyde under ambient conditions. Lang et al. [2] reported the synthesis of a thermally stable Pt single-atom catalyst dispersed on an Fe_2_O_3_ support. Experimental and computational modeling studies showed that the reducibility of the iron oxide is crucial for the anchoring of the isolated Pt atoms. Ren et al. [3] controlled the dispersion of platinum on Fe_2_O_3_ and CeO_2_ by using an ethanediamine chelate ligand and rapid thermal treatment. Liu et al. [4] used the co-precipitation method to disperse Pt on FeO_x_ support, while Li et al. [5] used the colloidal deposition method to prepare Pt/Fe_2_O_3_ catalysts for low-temperature oxidation CO to CO_2_ at room temperature. Li et al. [6] investigated the redispersion behavior of Pt on the surface of Fe_2_O_3_. They found that Pt can be effectively redispersed on the surface of Fe_2_O_3_ when it is alternately treated with oxidative and reductive atmosphere. The results of CO oxidation showed that the catalytic activities of Pt/Fe_2_O_3_ increased with the decrease of Pt particle size. D’Arienzo et al. [7] reported the one-step preparation of SnO_2_ and Pt-doped SnO_2_ in the form of inverted opal films obtained by sol-gel synthesis and dip coating. The electrical sensitivity of the inverted opal-based films was enhanced by Pt doping, and it was suggested that the increased porosity and electronic sensitization by the Pt dopant have a synergistic effect in improving the electrical response of the opal films. Dong et al. [8] reported a combustion method to prepare porous Pt-functionalized SnO_2_ sheets using urea as fuel. The synthesized Pt-SnO_2_ sheets exhibited better response and lower operating temperature than pristine SnO_2_ for gas detection of isopropanol gas.

Usually, the Pt dispersions on Fe_2_O_3_ and SnO_2_ metal oxide supports are prepared by wet impregnation or by sol-gel method using metal chloride precursors such as FeCl_3_, SnCl_4_ and H_2_PtCl_6_. In contrast, in this work, we use solid-state dispersion technique (mechanochemical) and compatible Fe(II)-acetate, Sn(II)-acetate and Pt(II)-acetylacetonate powders as organic precursors to better disperse Pt on Fe_2_O_3_ and SnO_2_ supports and avoid cation (sodium, potassium) [9] or anion (chloride) [10,11] contamination of the synthesized nanomaterial. It is expected that the exclusive use of the divalent metal cations (Fe(II), Sn(II) and Pt(II)) and strongly chelating organic ligands, such as acetate and acetylacetonate, will allow homogeneous mixing of the various M(II) precursors, limit crystal growth and lead to the uniform dispersion of the Pt catalyst on the metal oxide supports.

## 2. Materials and Methods

### 2.1. Chemicals and Synthesis of the Samples

Iron(II) acetate (Cat. No. 517933), tin(II) acetate (Cat. No. 345164), platinum(II) acetylacetonate (Cat. No. 282782) and anhydrous toluene (Cat. No. 244511) produced by Sigma-Aldrich, Burlington, MA, USA were used as received.

The pristine hematite (α-Fe_2_O_3_) **sample FE-0** was synthesized by grinding Fe(II) acetate in a planetary mill “FRITSCH PULVERISETTE 7 premium line”, Idar-Oberstein, Germany, with 40 balls of zirconia size 5 mm at a speed of 1000 rpm for two hours. After grinding, the orange-red powder was stored in a tube furnace for 30 min in an argon stream at 200 °C, 30 min in an argon stream at 400 °C, 1 h in an argon stream at 600 °C and 2 h in an air stream at 600 °C; a dark red powder was obtained, which was assigned as a **sample FE-0**.

The pristine tin oxide (cassiterite) **sample SN-0** was synthesized in the same manner as hematite sample FE-0, except that the starting chemical was tin(II) acetate instead of iron(II) acetate. A white powder was obtained and assigned as **sample SN-0**.

The platinum was dispersed in the SnO_2_ or Fe_2_O_3_ in three ways: *(*i) In one step, by mixing powdered Fe (II) acetate or Sn (II) acetate precursor with 1 mol% Pt(acac)_2_ and activation in a planetary mill, followed by annealing in a tube furnace for 30 min in an argon stream at 200 °C, 30 min in an argon stream at 400 °C, 1 h in an argon stream at 600 °C and 2 h in an air stream at 600 °C (**samples SN-1 and FE-1**). *(*ii) In two steps, where first hematite and cassiterite were synthesized at 600 °C, and then in the second step, these pristine powder samples were mixed with the 1 wt% Pt(acac)_2_ powder precursor and homogenized by grinding in a planetary mill at a speed of 400 rpm for 2 h using zirconia 5 mm balls. Subsequently, the homogenized powders were annealed in an argon stream for one hour and in an air stream for two hours at 400 °C (**samples SN-2 and FE-2**), (iii) In two steps, in which the hematite and the cassiterite were first synthesized at 600 °C, and then in the second step, the pristine powder samples were mixed with 1 wt% Pt(acac)_2_ previously dissolved in a certain volume of toluene, and then the paste obtained was homogenized in a planetary mill at a speed of 400 rpm for 2 h using zirconia 5 mm balls and annealed in an argon stream for one hour and in an air stream at 400 °C for two hours (**samples SN-3 and FE-3**).

### 2.2. Instrumental Analysis

X-ray diffraction (XRD) measurements were performed at room temperature using APD 2000 diffractometer (CuKα radiation, graphite monochromator, NaI-Tl detector) manufactured by ITALSTRUCTURES, Riva Del Garda, Italy.

Mössbauer spectra were collected in a transmission mode using standard instrumental configuration (WissEl GmbH, Starnberg, Germany). ^57^Co in rhodium matrix was used as a source of radiation. The spectrometer was calibrated at room temperature using a standard spectrum of α-Fe foil.

Atomic resolution scanning transmission electron microscope (AR STEM), model Jeol ARM 200 CF, with voltage emission of 200 kV coupled with Gatan Quantum ER system and with electron energy loss spectroscopy and energy dispersive X-ray spectrometry (Jeol Centurio 100), Tokyo, Japan was used.

Nitrogen adsorption measurements at 77 K for Brunauer–Emmett–Teller (BET) analysis and necessary degassing pre-treatment were conducted on Quantachrome Autosorb iQ3 system, Anton Paar QuantaTec 1900 Corporate Drive Boynton Beach, FL, USA.

Temperature-programmed reduction (TPR) experiments were performed using a Quantachrome Autosorb iQ3-AG-C instrument. In a typical experiment, 100 mg of sample was placed in a quartz tube, and the temperature was raised from room temperature to 120 °C in a stream of helium at a constant rate of 20 °C/min. The sample temperature was maintained at 120 °C for 40 min and then cooled to 40 °C. The sample was then heated to 800 °C under a reducing gas mixture (10% H_2_/90% Ar) at a rate of 10 °C/min. A thermal conductivity detector (TCD) was used to measure the changes in thermal conductivity of the effluent gas. At the end of the measurements, the samples were cooled in a stream of nitrogen.

X-ray photoelectron spectra (XPS) were recorded using a SPECS instrument (Berlin, Germany) under ultra-high vacuum (UHV) conditions (the typical pressure was in the range of 10^−7^ Pa) with an Al Kα X-ray of energy 1486.74 eV and the Phoibos100 electron energy analyzer. The transmission energy was set to 50 eV for the measurements around the Pt 4f core levels. Numerical fitting of the experimental spectra was performed using the mixed Gaussian–Lorentzian functions after Shirley background subtraction. All XPS spectra were corrected for their binding energy shifts using C 1s peaks at 284.2 eV as reference.

## 3. Results

**Figure 1:**
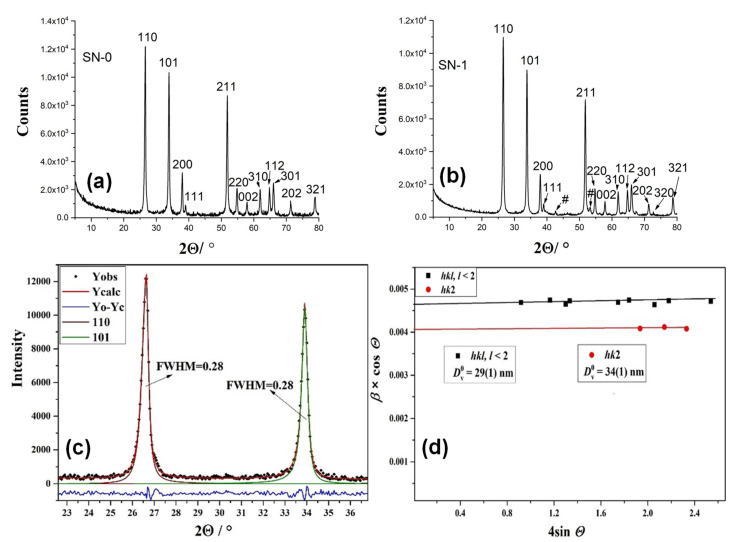
(**a**) XRD patterns of samples SN-0; (**b**) XRD patterns of samples SN-1. Both samples are assigned according to ICDD card 41-1445 (cassiterite). The hkl indices of cassiterite are indicated. # indicates unidentified maxima; (**c**) the results of individual profile fitting of cassiterite diffraction lines 110 and 101 in sample SN-0; (**d**) Williamson–Hall analysis of cassiterite in sample SN-0. The value of the volume-averaged domain size ($D_{v}^{0}$) was determined from the *y*-axis intercept and the value of the upper limit of microstrains (*e*), from the slope of the line.

Figure 1 shows the XRD patterns of samples SN-0 and SN-1. Both samples are assigned according to ICDD card 41-1445 (cassiterite, tetragonal space group P42/mnm). The hkl indices of cassiterite are given. Figure 1c shows the results of individual profile fitting of cassiterite diffraction lines 110 and 101 in sample SN-0. Figure 1d presents the Williamson–Hall analysis of sample SN-0. The value of the volume-averaged domain size ($D_{v}^{0}$) was obtained from the intercept of the *y*-axis, and the value of the upper limit of microstrains (*e*) was obtained from the slope of the line. The results of Williamson–Hall analysis showed the presence of anisotropy in sample SN-0, with slightly narrower diffraction lines with Miller indices hk2, indicating an increase in the size of the crystal domain (the values in the hk2 direction were estimated to ~34 nm). The XRD patterns, the results of individual profile fitting and Williamson–Hall analyses of samples SN-1 and SN-2 can be found in the Supplementary Materials.

**Figure 2:**
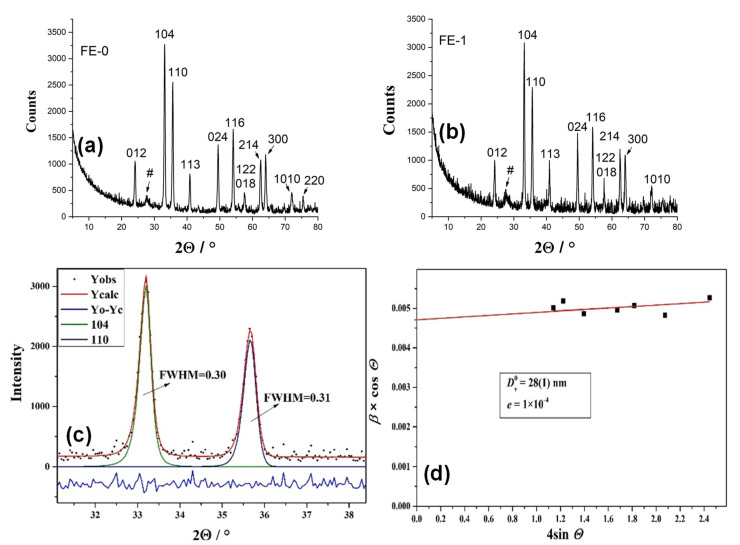
(**a**) XRD patterns of samples FE-0, # indicates unidentified maxima; (**b**) XRD patterns of samples FE-1. The both samples are assigned in accordance with the ICDD card 33-0664 (hematite). The hkl indices of hematite are indicated, # indicates unidentified maxima; (**c**) the results of individual profile fitting of the hematite diffraction lines 104 and 110 in the sample FE-1; (**d**) Williamson–Hall analysis of hematite in the sample FE-1. The value of the volume-averaged domain size ($D_{v}^{0}$) was obtained from the intercept on the *y*-axis, and the value of the upper limit of microstrains (*e*) from the slope of the line.

Figure 2 shows XRD results of samples FE-0 and FE-1. The patterns of both samples are assigned in accordance with the ICDD card 33-0664 (hematite, rhombohedral R-3c space group). The hkl indices of hematite are indicated (a, b), and the results of individual profile fitting of the hematite diffraction lines 104 and 110 in the sample FE-1 (c) and Williamson–Hall analysis of sample FE-1 (d) are shown. Sample FE-1 possessed a volume average crystal domain size of 28 nm with very small microstrains (the upper limit of microstrain was estimated to be ~1 × 10^−4^). The XRD patterns and line broadening analyses of samples FE-0, FE-2 and FE-3 can be found in the Supplementary Materials.

**Figure 3:**
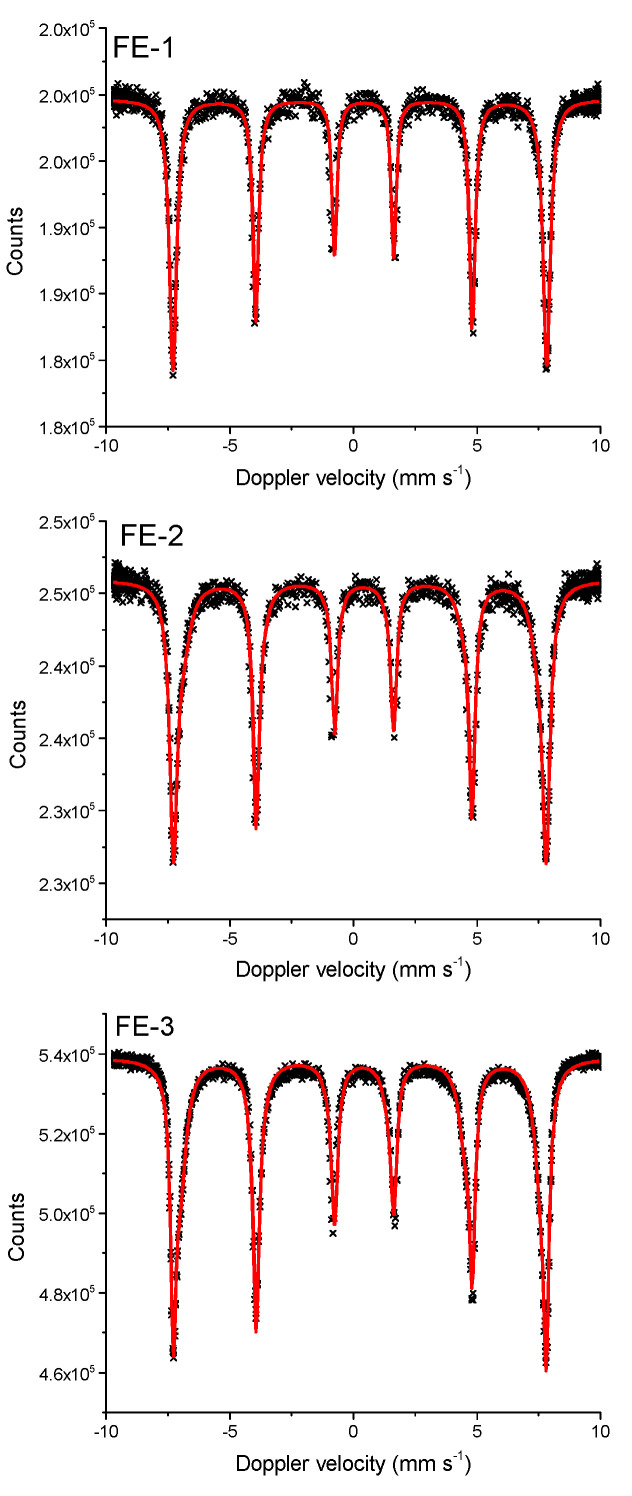
Mössbauer spectra of samples FE-1, FE-2 and FE-3 recorded at 20 °C.

Figure 3 shows the Mössbauer spectra of samples FE-1, FE-2 and FE-3 taken at 20 °C. The ^57^Fe Mössbauer parameters of the samples are listed in Table 1. All three samples are characterized with a sextet. The isomer shift (*δ*) of 0.35 mm s^−1^, the quadrupole shift of −0.17 mm s^−1^ and the hyperfine magnetic field (*B*_hf_) of ~46.89 mm s^−1^ can be attributed to hematite in agreement with the XRD results. The hyperfine magnetic field of well-crystallized hematite has a value of ~51.0 mm s^−1^ [12,13,14]. The *B*_hf_ and line width (*Γ*) are very sensitive to the crystallinity, particle size and surface properties of hematite [15,16,17,18]. In the present case, the *B*_hf_ decrease slightly, and *Γ* broadened from sample FE-1 to sample FE-3 (Table 1).

**Figure 4:**
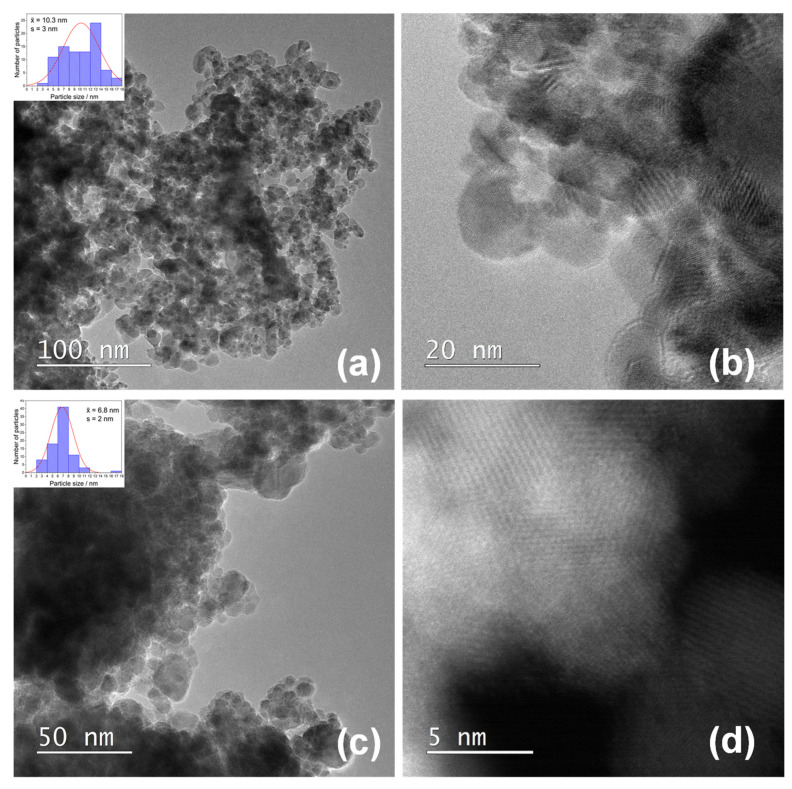
(**a**) STEM images and particle size distributions (inset) of samples SN-1 at low magnification and; (**b**) at high magnification; (**c**) STEM images and particle size distributions (inset) of samples SN-2 at low magnification and; (**d**) at high magnification.

Figure 4 shows STEM images of samples SN-1 (a) and SN-2 (c) at low magnification. Measurements of the particle size distributions (inset) gave mean values of 10.3 ± 3.4 and 6.8 ± 2.1 nm for samples SN-1 and SN-2, respectively. At high magnifications (b, d), the crystal planes are clearly visible, but there is no evidence of other (smaller) particles.

**Figure 5:**
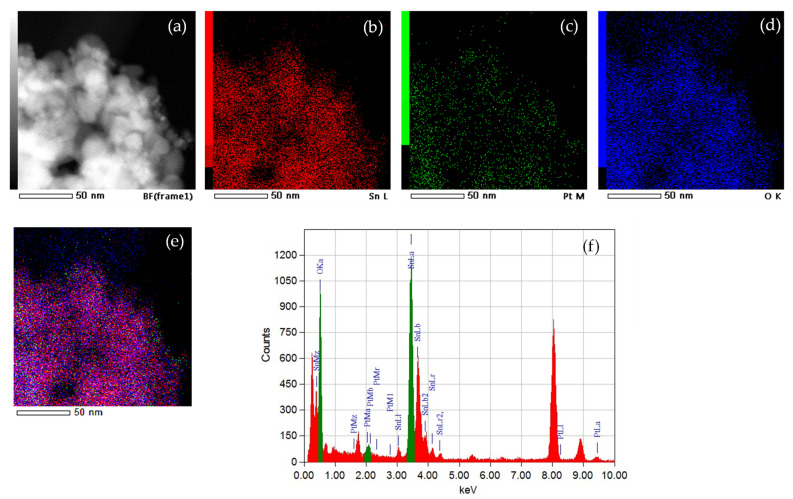
STEM image of sample SN-1 (**a**) and corresponding EDXS elemental mapping images of Sn L edge (**b**), Pt M edge (**c**), O K edge (**d**) and overlay of Sn L, Pt M and O K edges (**e**). EDXS spectrum of sample SN-1 (**f**).

Figure 5 shows the STEM image of sample SN-1 (a) and the corresponding EDXS elemental mapping images of Sn L edge (b), Pt M edge (c), O K edge (d) and overlay of the Sn L, Pt M and O K edges (e). The EDXS spectrum of sample SN-1 is also shown (f). It can be seen that all three elements are homogeneously dispersed and that there are no distinct Pt clusters. The STEM image and EDXS elemental mapping of sample SN-2 can be found in Figure S7 in the Supplementary Materials.

Figure S6 (Supplementary Materials) shows STEM images of samples FE-1 (a), FE-2 (b) and FE-3 (c). The mean particle sizes of 30.9, 20.9 and 8.6 nm were measured for samples FE-1, FE-2 and FE-3, respectively.

**Figure 6:**
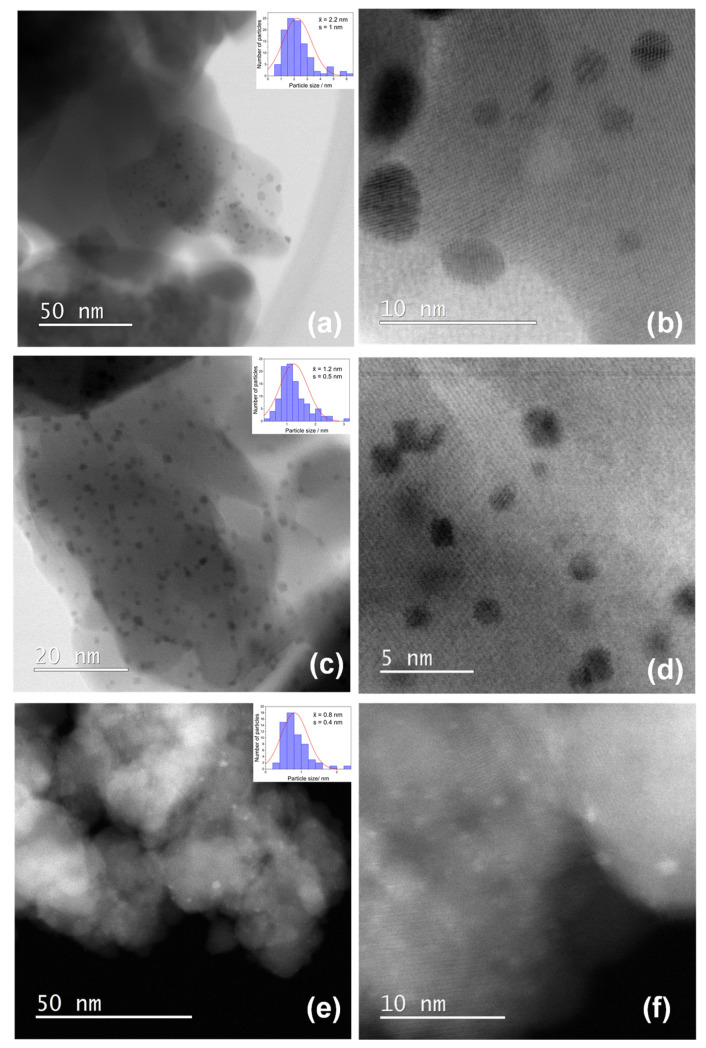
STEM bright-field images and particle size distributions (inset) of samples FE-1 at low magnification (**a**) and at high magnification (**b**); STEM bright-field images and particle size distributions (inset) of samples FE-2 at low magnification (**c**) and at high magnification (**d**); STEM dark-field images and particle size distributions (inset) of samples FE-3 at low magnification (**e**) and at high magnification (**f**).

Figure 6 shows STEM bright-field images with particle size distributions (inset) of small NPs in samples FE-1 (a, b) and FE-2 (c, d) and dark-field images with particle size distribution (inset) of very small NPs in sample FE-3 (e, f). One can see the well-dispersed small distinct NPs of 2.2, 1.2 and 0.8 nm in samples FE-1, FE-2 and FE-3, respectively. The 0.8 nm NPs in the FE-3 sample are so small that they are virtually invisible on the STEM bright-field image, so they are shown in dark-field mode.

**Figure 7:**
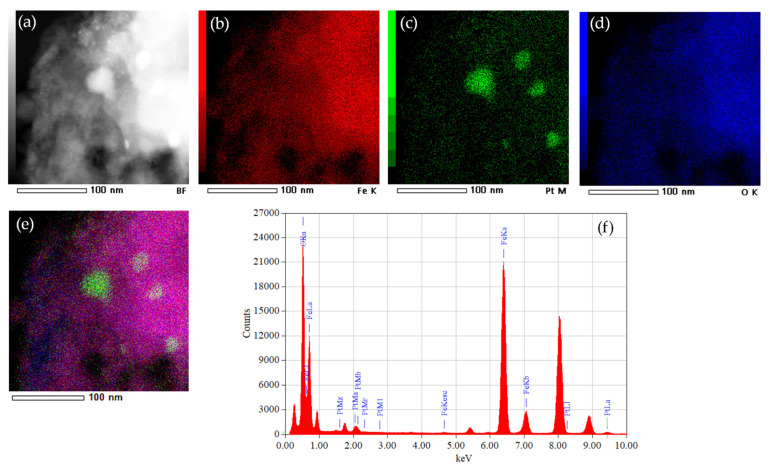
STEM image of sample FE-1 (**a**) and corresponding EDXS elemental mapping images of Fe K edge (**b**), Pt M edge (**c**), O K edge (**d**) and overlay of Fe K, Pt M and O K edges (**e**). EDXS spectrum of sample FE-1 (**f**).

Figure 7 shows the STEM image of sample FE-1 (a) and the corresponding EDXS elemental mapping images of the Fe K edge (b), Pt M edge (c), O K edge (d) and overlay of Fe K, Pt M and O K edges (e). The EDXS spectrum of sample FE-1 is shown (f). The individual Pt particles are clearly visible.

**Figure 8:**
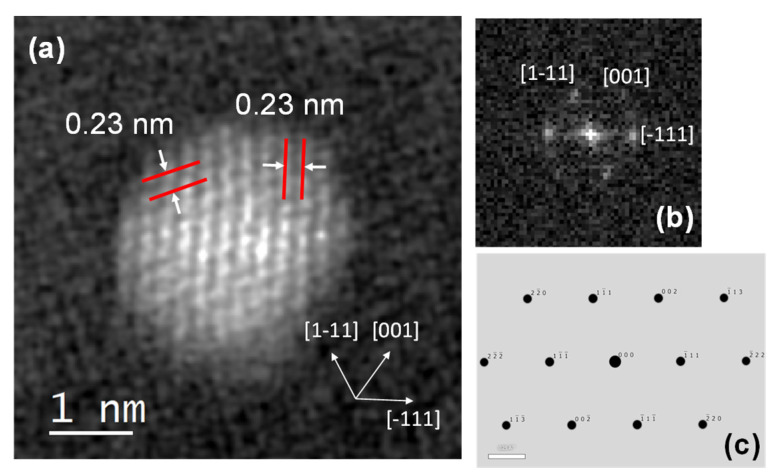
HAADF-STEM image of Pt nanoparticle oriented in [110] zone in sample FE-1 (**a**); FFT of HAADF-STEM image of Pt nanoparticle in [110] zone axis (**b**); simulated SAED pattern of cubic Pt in [110] zone (**c**).

Figure 8a shows high-angle annular dark-field scanning transmission electron microscopy (HAADF STEM) image of Pt nanoparticle oriented in [110] zone in sample FE-1. Lattice fringe analysis shows the average interplanar spacing of 0.23 nm corresponding to the planes of type {111} that go vertically and diagonally of face-centered cubic (FCC) platinum. Figure 8b shows the FFT of the HAADF-STEM image of a Pt nanoparticle in the [110] zone axis, and Figure 8c shows the simulated SAED pattern of cubic Pt in the [110] zone.

**Figure 9:**
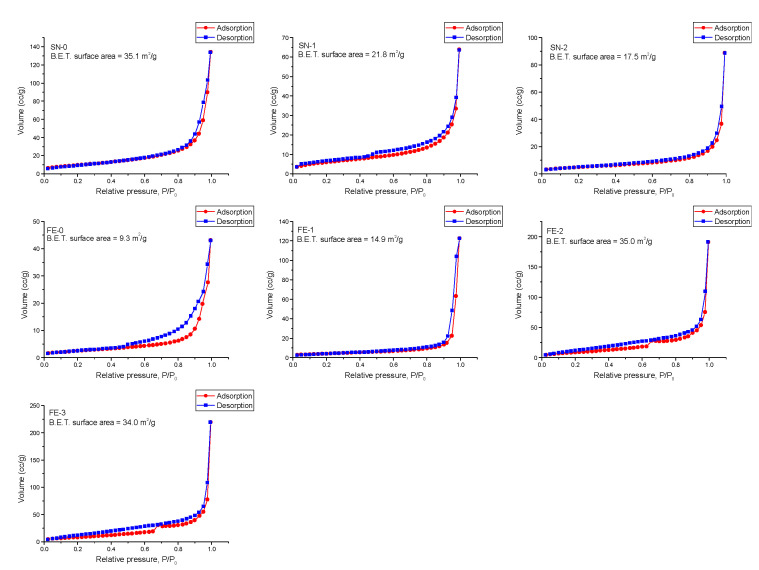
Gas (N2) adsorption (red line, circle) and desorption (blue line, squares) isotherms of samples SN-0, SN-1, SN-2, FE-0, FE-1, FE-2 and FE-3.

Figure 9 shows the gas (N2) adsorption (red line, circle) and desorption (blue line, squares) isotherms of the samples SN-0, SN-1, SN-2 and FE-0 to FE-3. Instead of a sample FE-0, where a loop of type H3 is observed, indicating slit-like pores of plate-like aggregated particles, the other samples show the reversible physisorption isotherms of type II. The calculated Brunauer, Emmett and Teller (BET) specific surface areas for the SN samples show a decrease in the BET surface area from 35.1 m^2^ g^−1^ in the SN-0 sample to 17.5 m^2^ g^−1^ in the SN-2 sample. The opposite effect occurs for the FE samples, where the BET surface area increases from 9.3 to 35 m^2^ g^−1^ for the FE-0 and FE-2 samples, respectively. The BET surface area of the FE-3 sample (34 m^2^ g^−1^) is very close to that of the FE-2 sample.

**Figure 10:**
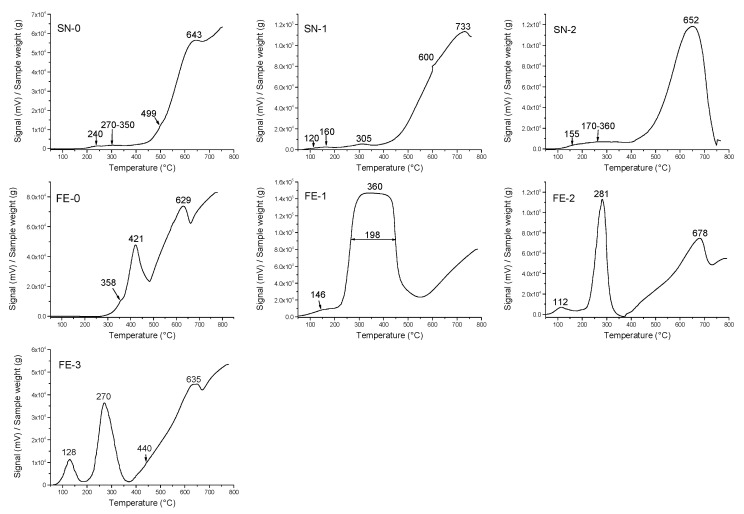
Temperature-Programmed Reduction in hydrogen (H_2_-TPR) results.

Figure 10 shows H_2_-TPR results. SN samples are relatively easily reduced with H_2_ according to the simplified equation SnO_2_ + 2 H_2_ → Sn + 2 H_2_O [19,20]. The H_2_–TPR curve of the pristine α-SnO_2_ (sample SN-0) showed that the reduction of SnO_2_ started at about 180 °C and ended with an “incomplete” maximum at 643 °C. The incomplete reduction of SnO_2_ to Sn can be attributed to the formation of the SnO and/or Sn metal shell around the α-SnO_2_ particles. The melting point of tin is very low at 232 °C, so molten tin on the surface of α-SnO_2_ particles can protect Sn^4+^ from reduction. The addition of Pt promotes the reduction of α-SnO_2_, and the reduction started at a lower temperature (~100 °C) and ended with a well-defined maximum at 652 or 733 °C for samples SN-2 and SN-1, respectively.

In the pristine α-Fe_2_O_3_ (sample FE-0), the reduction starts at 300 °C and shows two peaks at 421 and 629 °C. The first maximum at 421 °C corresponds to the reduction of α-Fe_2_O_3_ → Fe_3_O_4_, and the second maximum at 629 °C to the reduction of Fe_3_O_4_ → FeO → Fe [6]. Doping with platinum in one step (sample FE-1) significantly increases the reduction of α-Fe_2_O_3_, two reduction maxima at 421 and 629 °C integrate a broad maximum at 360 °C, and a new small and very broad reduction maximum at 146 °C can be attributed to Pt^4+^ reduction in Pt^0^ and partial Fe^3+^ reduction ions bound in Fe-O-Pt groups. When Pt is doped in two steps (samples FE-2 and FE-3), two reduction maxima are again present, with the maximum shifted to lower temperatures (281 and 270 °C) due to α-Fe_2_O_3_ → Fe_3_O_4_ reduction, while Fe_3_O_4_ → FeO → Fe reduction is shifted to higher reduction temperatures (678 °C), and the maximum is at a lower temperature at 635 °C for sample FE-3. For the two-step platinum doping, the Pt^4+^ → Pt^0^ reduction maxima are well defined and distinct at 112 and 128 °C for samples FE-2 and FE-3, respectively. In the pristine α-Fe_2_O_3_ (sample FE-0) and α-SnO_2_ (sample SN-0) there is no platinum, and consequently, there is no reduction maxima below 200 °C.

**Figure 11:**
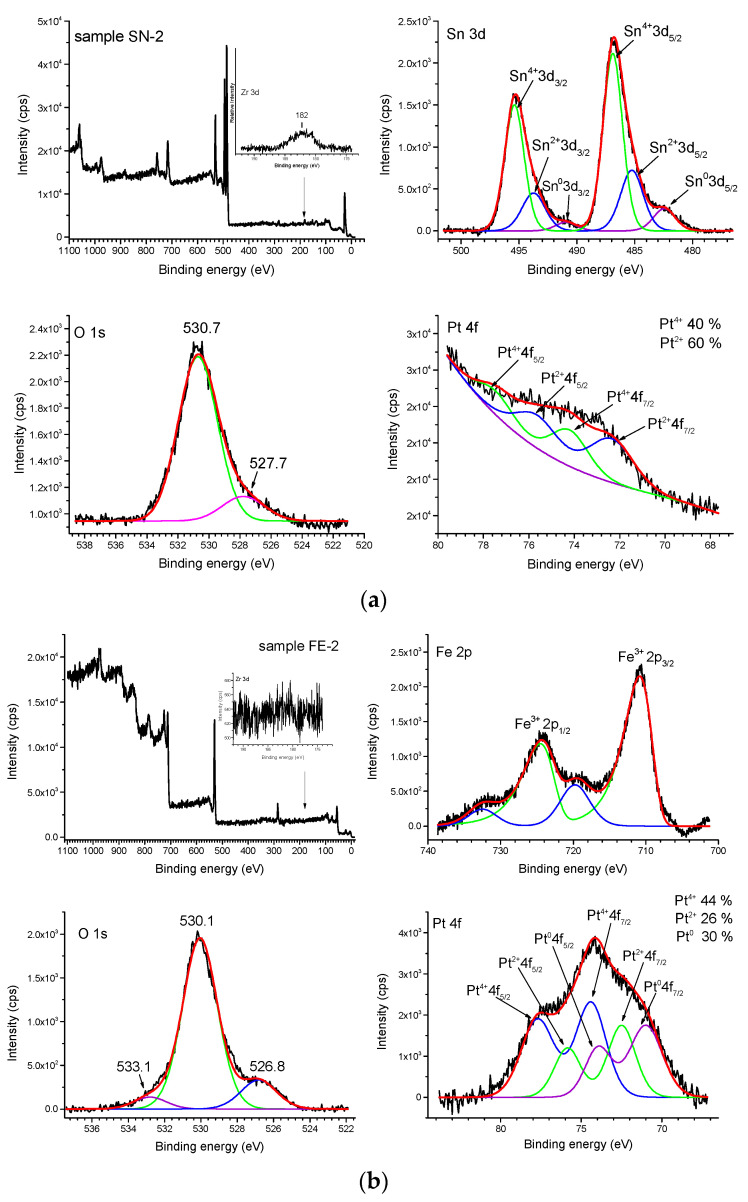
XPS spectra of samples SN-2, panel (**a**) and sample FE-2, panel (**b**).

Figure 11 shows the results of XPS analysis for samples SN-2 (a) and sample FE-2 (b). The presence of small Zr impurities in the samples due to the ball milling process (ZrO_2_ balls) is shown in the inset of the XPS survey scanning spectrum of the samples SN-2 and FE-2. The high-resolution XPS spectra of Sn 3d, O 1s, and Pt 4f of sample SN-2 are shown in panel (a). The binding energies of Sn 3d_3/2_ and Sn 3d_5/2_ at 495.4 and 486.9 eV, respectively, are attributed to Sn^4+^, at 493.7 and 485.2 eV to Sn^2+^, and at 490.8 and 482.5 eV to Sn^0^ [21,22], which is consistent with the NIST X-ray photoelectron spectroscopy database. The binding energy of O1s at 531 eV (530.9 eV in Table 2) can be assigned to the O^2−^ of lattice oxygen. The peaks of Pt 4f are rather weak, which can be attributed to the low concentration of Pt in the samples. The Pt 4f peak was fitted without background subtraction. XPS peaks Pt 4f_5/2_ and Pt 4f_7/2_ at 77.6 and 74.1 eV, respectively, are attributed to Pt^4+^ and at 75.8 and 72.3 eV, to Pt^2+^ (Table 2) [23,24,25].

Figure 11, panel (b) shows the high-resolution Fe 2p spectrum. The two distinct peaks at binding energies of 710.8 eV for Fe 2p_3/2_ and 724.7 eV for 2p_1/2_ are consistent with Fe^3+^ in α-Fe_2_O_3_ [26]. In addition, two satellite peaks characteristic of α-Fe_2_O_3_ at 719.8 eV and 732.5 eV are clearly visible [22,25]. The O 1s peak consists of three deconvoluted peaks at 526.8, 530.1, and 533.1 eV (Table 1). The binding energy of O1s at 530.1 eV can be associated with O^2−^ lattice oxygen species, while the peaks at 526.8 eV are most likely related to surface defects (highly oxidative oxygen species $O_{2}^{2-}$, O^−^ associated with oxygen vacancies, Fe-O-Pt surface bonds, etc.). The peak at 533.1 eV can be attributed to adsorbed oxygen species (e.g., hydroxyl groups -OH, water molecules H_2_O and/or surface-adsorbed oxygen O_2_). The binding energies of Pt 4f_7/2_ and Pt 4f_5/2_ were fitted to Pt^0^ (70.9 and 73.9 eV), Pt2+ (72.5 and 75.9 eV) and Pt^4+^ (74.4 and 77.9 eV). The percentage of Pt^0^, Pt^2+^ and Pt^4+^ was 30, 26 and 44%, respectively.

## 4. Discussion

The dispersion of platinum (Pt) on metal oxide supports is important for catalytic and gas sensing applications. Usually, Pt is dispersed by a wet impregnation method starting from H_2_PtCl_6_ (chloroplatinic acid) or K_2_PtCl_6_ (potassium hexachloroplatinate). In contrast, in this work, we mechanochemically dispersed Pt in nanocrystalline α-Fe_2_O_3_ and α-SnO_2_ starting from compatible Fe(II) acetate, Sn(II) acetate and Pt(II) acetylacetonate powders. In a one-step procedure, the Pt(acac)_2_ powder was mixed with Fe(II) acetate or Sn(II) acetate powder and homogenized in a planetary mill. Subsequently, the samples were annealed at 600 °C. The dispersion of platinum over α-SnO_2_ is clearly different from the Pt dispersion over α-Fe_2_O_3_. Figure 4a shows STEM images of the samples SN-1 with the 10.3 nm nanoparticles, while EDXS analysis (Figure 5) confirms that these nanoparticles belong to SnO_2_ and that there are no isolated Pt clusters. The Sn, O and Pt elements are homogeneously dispersed, strongly suggesting that the platinum is incorporated into the α-SnO_2_ structure. In contrast, the STEM image and EDXS analysis of the sample FE-1 (Figure 7a,b and Figure 8) show the well-dispersed isolated Pt nanoparticles with a size of 2.2 nm. Moreover, the size of the Pt nanoparticles could be controlled by changing the experimental conditions. In the two-step procedure, mixing the synthesized pure α-Fe_2_O_3_ powder (sample FE-0) with Pt(acac)_2_ powder in the planetary mill and then annealing at 400 °C resulted in smaller, well-dispersed, discrete Pt nanoparticles with a size of 1.2 nm. When the Pt(acac)_2_ powder was previously dissolved in a certain volume of toluene and then mixed with pure α-Fe_2_O_3_ in a planetary mill, the obtained PtNPs are 0.8 nm in size. In the case of SnO_2_ samples, there were no isolated PtNPs regardless of the experimental conditions. The unexpectedly different Pt dispersion over α-SnO_2_ and α-Fe_2_O_3_ reducible metal oxide supports could be explained by the different chemistry and oxidation state of Sn^4+^ and Fe^3+^ cations. The Sn^4+^ cation is easily reducible and can be easily reduced to metallic state Sn^0^ [27], as shown by the TPR and XPS results (Figure 10 and Figure 11). Moreover, the TPR results (Figure 10) showed that Pt promotes the reduction of Sn^4+^. It is known that SnPt nanoparticles can be synthesized from the same organic precursors used in this work, i.e., Sn(II) acetate and Pt(II) acetylacetonate by a polyalcohol reduction in the liquid phase [28]. However, every attempt to find Pt or SnPt in SnO_2_ samples was unsuccessful. For instance, the HAADF image of sample SN-1 shows a uniform contrast, whereas Pt nanoparticles and clusters are clearly visible in sample FE-1 (Figure S7 in the Supplementary Materials). Sn and Pt have very different atomic numbers (50 vs. 78), and the intensity of the HAADF image (besides the thickness) is approximately related to Z^1.7^. In the case of Pt-based particles or Pt-rich surface layers, this should be seen as areas of higher contrast. On the other hand, the ionic radii of Fe^3+^ (0.645 Å), Sn^4+^ (0.690 Å) and Pt^4+^ (0.625 Å) in octahedral coordination are similar, and formally, Pt^4+^ could be incorporated as the smallest cation in the crystal lattice of α-SnO_2_ and α-Fe_2_O_3_. However, Pt^4+^ can be stabilized in the α-SnO_2_ structure by replacing Sn^4+^ with Pt^4+^ in the crystal lattice, resulting in a (Pt,Sn)O_2_ solid solution, while the substitution of Fe^3+^ with Pt^4+^ is unfavorable and Pt^4+^ is mainly ejected as Pt^0^ from α-Fe_2_O_3_ during annealing. Anenburg et al. [29] showed that Pt^4+^ doping of hematite was only possible at high pressure, which allowed the oxygen fugacity to be maintained at sufficiently high values to stabilize Pt^4+^ in hematite. In contrast, Murata et al. [30] have shown that Pt^4+^ is located at the Sn^4+^ site of the α-SnO_2_ lattice, thus forming a (Pt,Sn)O_2_ solid solution at Pt loading up to 10 at%. Our solid-state dispersion of platinum is therefore in agreement with the references [29,30] and showed that the cationic platinum (Pt^4+^) was homogeneously dispersed in the α-SnO_2_ structure under oxidative annealing (Figure 5), whereas in Fe_2_O_3_, despite the oxidative conditions, Pt^4+^ was expelled from the α-Fe_2_O_3_ crystal lattice and dispersed mainly as isolated platinum nanoparticles over α-Fe_2_O_3_ (Figure 7 and Figure 8).

## 5. Conclusions

Solid-state dispersion and compatible Fe(II) actetate, Sn(II) acetate and Pt(II) acetylacetonate powders as organic precursors were used to avoid cationic and anionic impurities and disperse Pt in nanocrystalline hematite (α-Fe_2_O_3_) and cassiterite (α-SnO_2_).

EDXS results (Figure 5) have shown that Sn, O and Pt elements are homogeneously dispersed in α-SnO_2_, indicating the formation of a (Pt,Sn)O_2_ solid solution.

Platinum is dispersed in α-Fe_2_O_3_ mainly in the form of isolated Pt nanoparticles (Figure 7 and Figure 8). The size of the dispersed Pt nanoparticles over α-Fe_2_O_3_ can be controlled by changing the experimental conditions and is set to 2.2, 1.2 and 0.8 nm (Figure 7).

The rather different Pt dispersion in α-SnO_2_ and α-Fe_2_O_3_ is due to the fact that Pt^4+^ can be stabilized in the α-SnO_2_ structure by replacing Sn^4+^ with Pt^4+^ in the crystal lattice, while the substitution of Fe^3+^ with Pt^4+^ is unfavorable, and Pt^4+^ is mainly expelled from the lattice at the surface of α-Fe_2_O_3_ to form isolated platinum nanoparticles despite the oxidative conditions during annealing.

## Figures and Tables

**Table 1:** ^57^Fe Mössbauer parameters of samples FE-1, FE-2 and FE-3 recorded at 20 °C.

Sample	Fitting Curve	*δ*/ mm s^−1^	2*ε/*mm s^−1^	*B*_hf_*/* T	*Γ/* mm s^−1^	*χ* ^2^
FE-1	M1	0.35	−0.17	46.99	0.30	2.78
FE-2	M1	0.35	−0.17	46.89	0.40	2.02
FE-3	M1	0.35	−0.16	46.58	0.41	2.15

Key: M1 = Magnetic component fitted to Mössbauer sextet; *δ* = isomer shift given relative to α-Fe at 20 °C; 2*ε* = quadrupole shift; *B*_hf_ = hyperfine magnetic field; *Γ* = line width. Error: *δ* = ±0.01 mm s^−1^; 2*ε* = ±0.01 mm s^−1^; *B*_hf_ = ±0.2 T; *χ*^2^ = goodness of fit.

**Table 2:** Peak positions of deconvoluted Pt 4f, Fe 2p, O 1s and Sn 3d XPS spectra of samples SN-2 and FE-2.

**Sample**	**Pt 4f/(eV)**	**Sn 3d/(eV)**	**O 1s/(eV)**
**SN-2**	Pt^4+^ 4f_7/2_/74.1	Sn^4+^ 3d_5/2_/486.9	O 1s/530.7
	Pt^4+^ 4f_5/2_/77.6	Sn^4+^ 3d_3/2_/495.4	O 1s/527.7
	Pt^2+^ 4f_7/2_/72.3	Sn^2+^ 3d_5/2_/485.2	
	Pt^2+^ 4f_5/2_/75.8	Sn^2+^ 3d_3/2_/493.7	
		Sn^0^ 3d_5/2_/482.5	
		Sn^0^ 3d_3/2_/490.8	
**Sample**	**Pt 4f/(eV)**	**Fe 2p/(eV)**	**O 1s/(eV)**
**FE-2**	Pt^4+^ 4f_7/2_/74.4	Fe^3+^ 3p_3/2_/710.8	O 1s/526.8
	Pt^4+^ 4f_5/2_/77.9	Fe^3+^ 3p_1/2_/724.7	O 1s/530.1
	Pt^2+^ 4f_7/2_/72.5	Satellite peak/719.8	O 1s/533.1
	Pt^2+^ 4f_5/2_/75.9	Satellite peak/732.5	
	Pt^0^ 4f_7/2_/70.9		
	Pt^0^ 4f_5/2_/73.9		

## Data Availability

The data presented in this study are available on request from the corresponding author.

## Supplementary Materials

The following are available online at https://www.mdpi.com/article/10.3390/nano11123349/s1, XRD individual profile fitting and Williamson–Hall analysis (Figures S1–S5), XRD line broadening analysis (Tables S1–S10) and TEM with particle size distributions, HAADF images and EDXS elemental mapping of selected samples (Figures S6–S8).
