# Supplementary material for: Solid-State Dispersions of Platinum in the SnO2 and Fe2O3 Nanomaterials
Source: Nanomaterials (Basel). 2021 Dec 10;11(12):3349. doi: 10.3390/nano11123349 (PMC8704069; doi:10.3390/nano11123349)
Supplement: Supplementary file 1 [file nanomaterials-11-03349-s001.zip › nanomaterials-1463754-supplementary.pdf]

Supplementary Materials

Solid state dispersions of platinum in the SnO_2 and Fe_2O_3 nanomaterials

Edi Radin [1], Goran Štefanić [1], Goran Dražić [2,*], Ivan Marić [3], Tanja Jurkin [3], Anđela Pustak [3], Nikola Baran [1], Matea Raić [1] and Marijan Gotić [1,*]

[1] Laboratory for Molecular Physics and Synthesis of New Materials, Ruđer Bošković Institute, Bijenička c. 54, 10000 Zagreb, Croatia; Edi.Radin@irb.hr (E.R.); Goran.Stefanic@irb.hr (G.Š.); nikola.baran@irb.hr (N.B.); matea.raic@irb.hr (M.R.)
[2] National Institute of Chemistry, Hajdrihova 19, SI-1001 Ljubljana, Slovenia
[3] Radiation Chemistry and Dosimetry Laboratory, Ruđer Bošković Institute, Bijenička c. 54, 10000 Zagreb, Croatia; imaric@irb.hr (I.M.); tjurkin@irb.hr (T.J.); Andjela.Pustak@irb.hr (A.P.)
* Correspondence: gotic@irb.hr (M.G.); goran.drazic@ki.si(G.D.)

Table of contents

1) **XRD individual profile fitting and Williamson-Hall analysis (Figures S1–S5)**
2) **XRD line broadening analysis (Tables S1–S10)**
3) **TEM with particle size distributions, HAADF images and EDXS elemental mapping of selected samples (Figures S6–S8)**
4) **References**

1) XRD individual profile fitting and Williamson-Hall analysis (Figure S1 to S5)

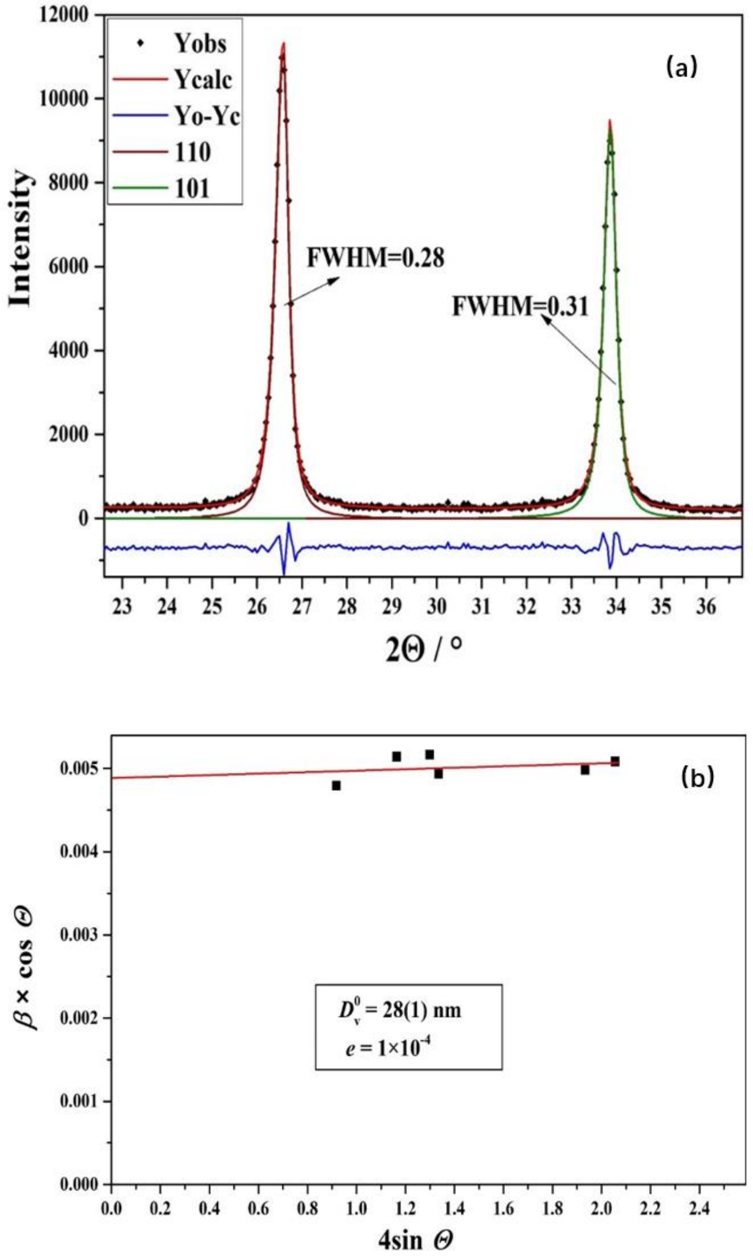

Figure S1. (**a**) The results of individual profile fitting of the cassiterite diffraction lines 110 and 101 in the **sample SN-1** (program XFIT). The differences between the observed (Y_{obs}) and calculated (Y_{cal}) patterns are shown in the box below. (**b**) Williamson-Hall analysis of hematite phase in the **sample SN-1**. The value of the volume-averaged domain size (D_v^0) was obtained from the intercept on the y-axis, and the value of the upper limit of microstrains (*e*) from the slope of the line.

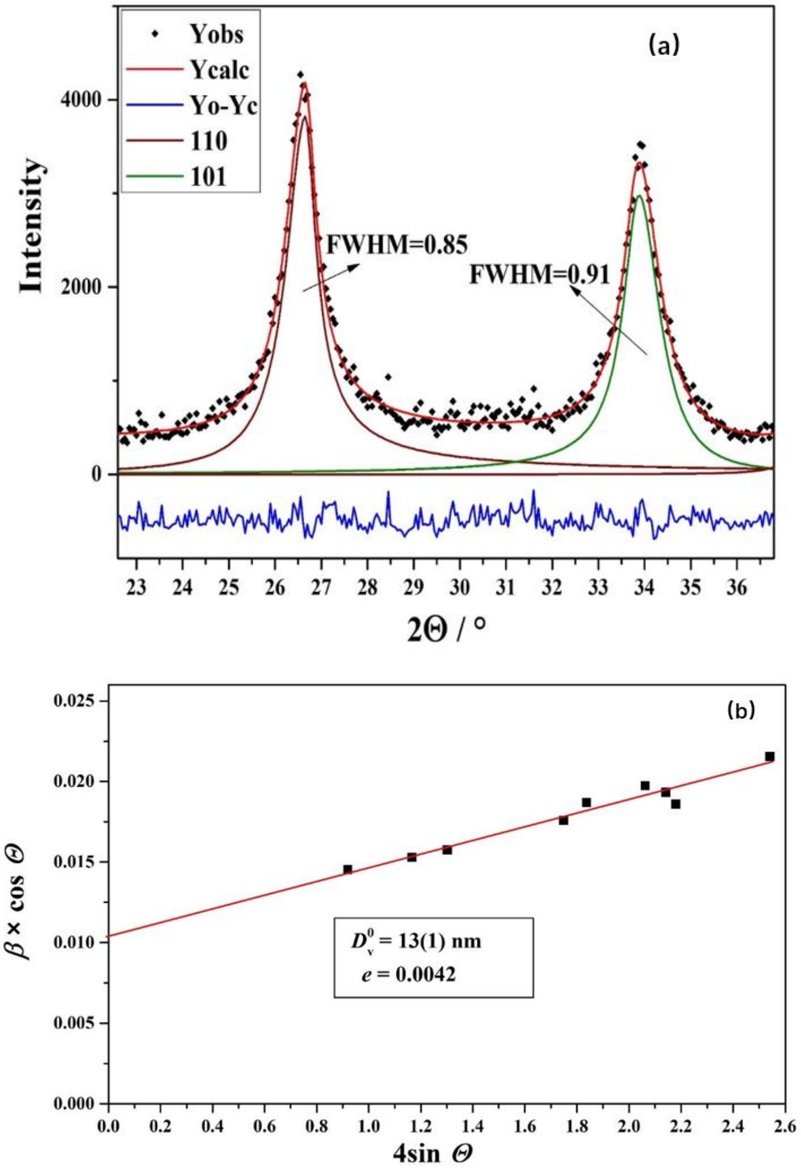

Figure S2. (**a**) The results of individual profile fitting of the cassiterite diffraction lines 110 and 101 in the **sample SN-2** (program XFIT). The differences between the observed (Y_{obs}) and calculated (Y_{cal}) patterns are shown in the box below. (**b**) Williamson-Hall analysis of hematite phase in the **sample SN-2**. The value of the volume-averaged domain size (D_v^0) was obtained from the intercept on the y-axis, and the value of the upper limit of microstrains (*e*) from the slope of the line.

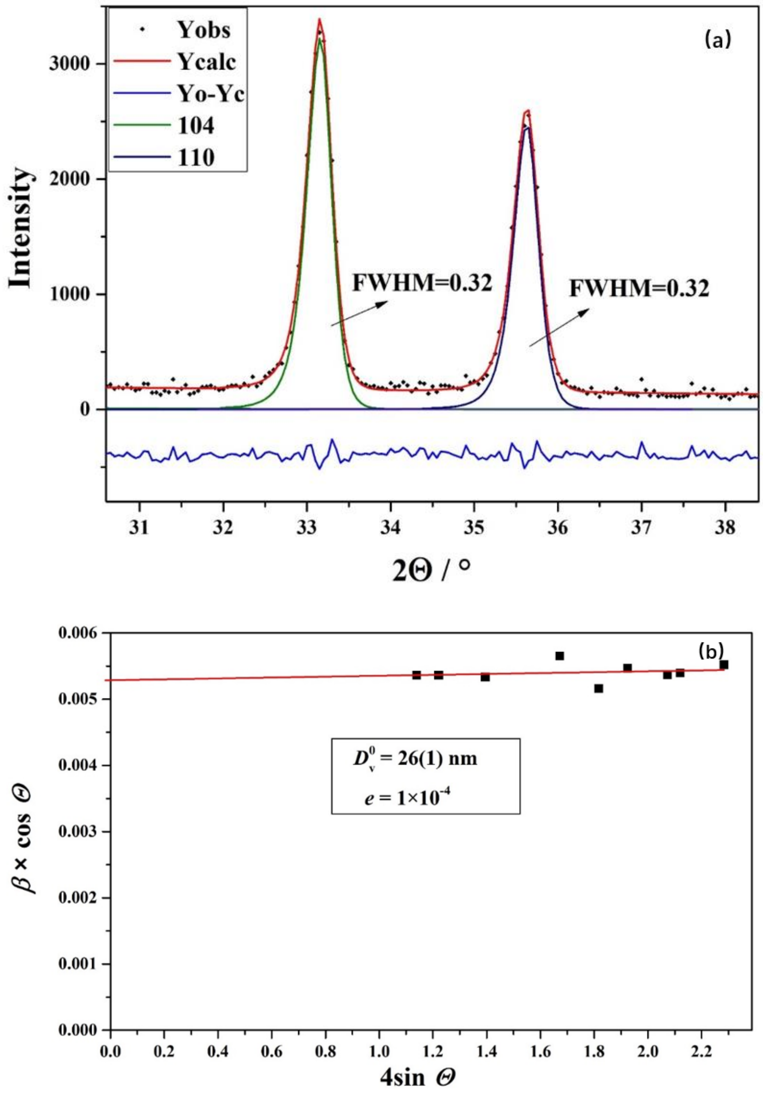

Figure S3. (**a**) The results of individual profile fitting of the hematite diffraction lines 104 and 110 in the **sample FE-0** (program XFIT). The differences between the observed (Y_{obs}) and calculated (Y_{cal}) patterns are shown in the box below. (**b**) Williamson-Hall analysis of hematite phase in the **sample FE-0.** The value of the volume-averaged domain size (D_v^0) was obtained from the intercept on the y-axis, and the value of the upper limit of microstrains (*e*) from the slope of the line.

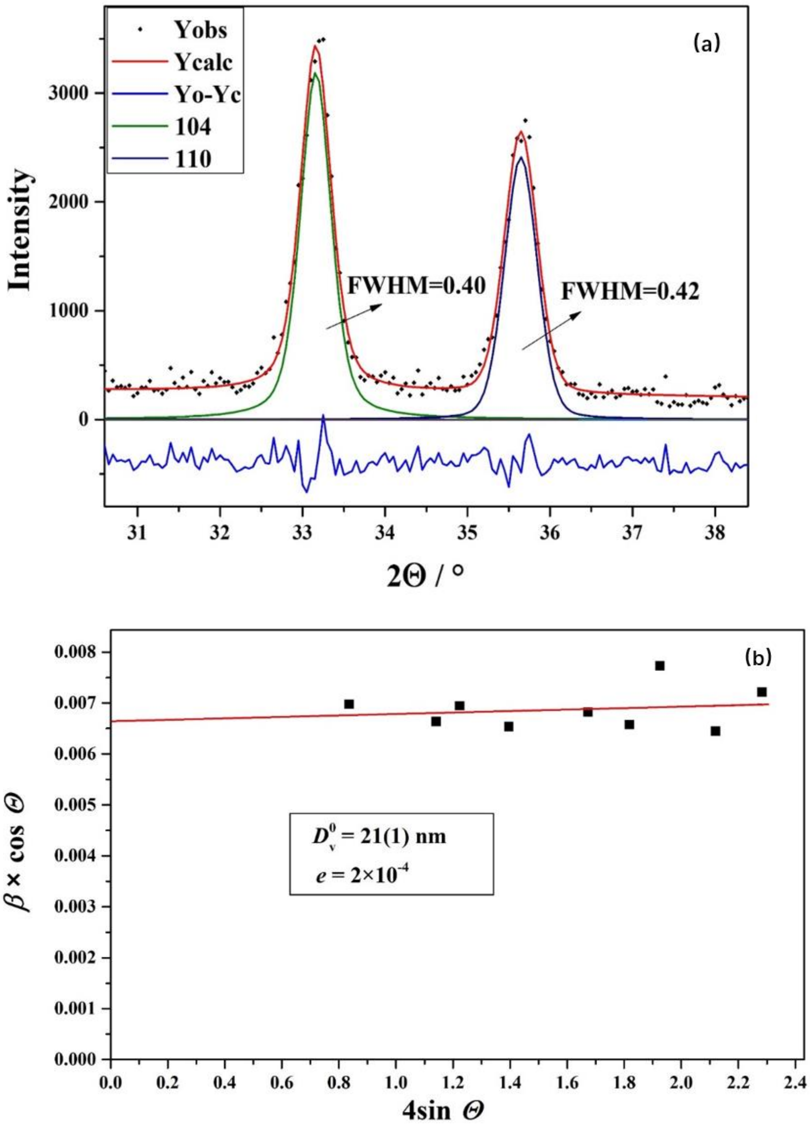

Figure S4. (**a**) The results of individual profile fitting of the hematite diffraction lines 104 and 110 in the **sample FE-2** (program XFIT). The differences between the observed (Y_{obs}) and calculated (Y_{cal}) patterns are shown in the box below. (**b**) Williamson-Hall analysis of hematite phase in the **sample FE-2**. The value of the volume-averaged domain size (D_v^0) was obtained from the intercept on the y-axis, and the value of the upper limit of microstrains (*e*) from the slope of the line.

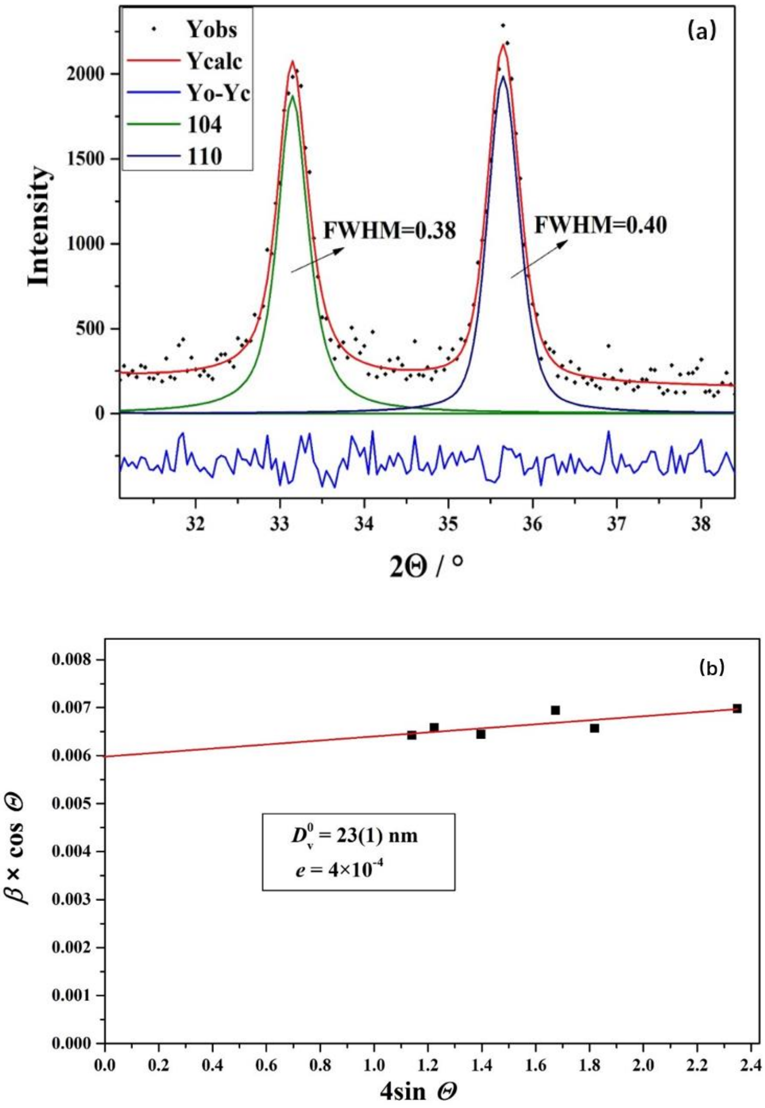

Figure S5. (**a**) The results of individual profile fitting of the hematite diffraction lines 104 and 110 in the **sample FE-3** (program XFIT). The differences between the observed (Y_{obs}) and calculated (Y_{cal}) patterns are shown in the box below. (**b**) Williamson-Hall analysis of hematite phase in the **sample FE-3**. The value of the volume-averaged domain size (D_v^0) was obtained from the intercept on the y-axis, and the value of the upper limit of microstrains (*e*) from the slope of the line.

2. XRD line broadening analysis

Volume average domain size in the direction normal to the reflecting planes *hkl* were estimated from the Scherrer equation:

$$D_{hkl} = \frac{0.9\lambda}{\beta_{hkl} \times \cos\theta}, \quad (1)$$

where D_{hkl} is a volume average domain size in the direction normal to the reflecting planes *hkl*, λ is the x-ray wavelength (Cu$K\alpha$), θ is the Bragg angle and β_{hkl} is the pure full width of the diffraction line (*hkl*) at half the maximum intensity. β_{hkl} values were determined from full-width at half the maximum intensity of diffraction lines (B_{hkl}), after correction for instrumental broadening for which the appropriate diffraction line width of a well-crystalline ZnO sample was used [1]. B_{hkl} values were obtained from the results of individual profile fitting using the program XFIT [2].

Separation of the influence of crystal size on the diffraction line broadening from the influence of defects within the crystal lattice was performed using the results of Williamson-Hall analysis [3]:

$$\left(\frac{\beta_{hkl} \times \cos\theta}{\lambda}\right) = \frac{K}{D_v^0} \times \left(\frac{4e \times \sin\theta}{\lambda}\right), \quad [1]$$

where D_v^0 is volume-averaged domain size and *e* stands for the upper-limits of microstrains.

Table S1. Sample names and corresponding phase composition.

SAMPLE	PHASE COMPOSITION
FE-0	Hematite (α-Fe_2O_3)
FE-1	Hematite (α-Fe_2O_3)
FE-2	Hematite (α-Fe_2O_3)
FE-3	Hematite (α-Fe_2O_3)
SN-0	Cassiterite (α-SnO_2)
SN-1	Cassiterite (α-SnO_2)
SN-2	Cassiterite (α-SnO_2)

Table S2. Results of individual profile fitting of the hematite phase in the FE-0 sample (program XFIT), and the corresponding D_{hkl} values calculated from the Scherrer equation.

hkl	Area	2 Θ	Lortz.	FWHM	D_{hkl}
0 1 2	379,42	24,07	0,1549	0,360	23
1 0 4	1408,29	33,10	0,3653	0,320	26
1 1 0	1107,35	35,58	0,3923	0,322	26
1 1 3	328,10	40,82	0,3541	0,326	26
0 2 4	591,54	49,43	0,233	0,356	25
1 1 6	799,51	54,04	0,5186	0,331	27
2 1 1	52,08	55,64	0,9036	0,308	25
1 2 2	234,52	57,53	0,954	0,357	26
2 1 4	568,92	62,42	0,4887	0,359	26
3 0 0	604,12	63,99	0,5312	0,364	25
2 0 8	41,81	69,60	0	0,385	23

Table S3. Results of individual profile fitting of the hematite phase in the FE-1 sample (program XFIT), and the corresponding D_{hkl} values calculated from the Scherrer equation.

hkl	Area	2 Θ	Lortz.	FWHM	D_{hkl}
0 1 2	309,58	24,11	0,0391	0,352	23
1 0 4	1188,52	33,15	0,3371	0,299	27
1 1 0	860,68	35,61	0,2174	0,312	27
1 1 3	287,42	40,87	0,2681	0,297	28
0 2 4	517,44	49,47	0,3794	0,312	28
1 1 6	657,87	54,07	0,2665	0,325	27
2 1 4	519,92	62,47	0,4564	0,323	29
3 0 0	514,79	64,02	0,5438	0,323	29
2 2 0	81,92	75,46	0	0,381	26

Table S4. Results of individual profile fitting of the hematite phase in the FE-2 sample (program XFIT), and the corresponding D_{hkl} values calculated from the Scherrer equation.

hkl	Area	2 Θ	Lortz.	FWHM	D_{hkl}
0 1 2	720,23	24,13	0,6173	0,408	20
1 0 4	1819,03	33,16	0,5317	0,396	21
1 1 0	1310,79	35,64	0,2485	0,417	20

1 1 3	417,06	40,88	0,3561	0,399	21
0 2 4	761,62	49,50	0,7985	0,430	20
1 1 6	1064,48	54,13	0,8588	0,422	21
0 1 8	196,29	57,60	0,6064	0,505	18
3 0 0	705,93	64,06	0,7267	0,435	22
2 0 8	66,47	69,69	0,0234	0,503	19

Table S5. Results of individual profile fitting of the hematite phase in the FE-3 sample (program XFIT), and the corresponding D_{hkl} values calculated from the Scherrer equation.

hkl	Area	2 Θ	Lortz.	FWHM	D_{hkl}
0 1 2	313.7852	24,10	0,2675	0,423	19
1 0 4	1184.2091	33,12	0,8164	0,383	22
1 1 0	1129.1106	35,62	0,4814	0,396	21
1 1 3	329.929	40,88	0,9191	0,394	22
0 2 4	560.6426	49,48	1	0,438	20
1 1 6	755.9283	54,08	1	0,422	21
0 1 8	237.5661	57,46	0,7243	0,526	17
3 0 0	396.3072	64,04	0,4953	0,400	23
1 0 10	140.66	71,92	0,0006	0,493	20

Table S6. Results of individual profile fitting of the cassiterite phase in the SN-1 sample (program XFIT), and the corresponding D_{hkl} values calculated from the Scherrer equation.

hkl	Area	2 Θ	Lortz.	FWHM	D_{hkl}
1 1 0	5474,616	26,5253	0,6804	0,282	29
1 0 1	4433,786	33,8221	0,7181	0,308	27
2 0 0	1212,69	37,906	0,689	0,313	27
1 1 1	430,7164	38,9908	0,9999	0,303	28
2 1 1	3651,274	51,7491	0,7637	0,290	30
2 2 0	793,2979	54,7373	0,602	0,303	30
0 0 2	430,5814	57,8083	0,7944	0,326	28
3 1 0	763,8534	61,8649	0,613	0,339	27
1 1 2	792,7732	64,7272	0,7203	0,298	31
3 0 1	1158,9	65,9645	0,872	0,327	29
2 0 2	395,519	71,2562	0,7759	0,320	31
3 2 0	23,3448	72,7145	0	0,347	30

Table S7. Results of individual profile fitting of the cassiterite phase in the SN-2 sample (program XFIT), and the corresponding D_{hkl} values calculated from the Scherrer equation.

hkl	Area	2 Θ	Lortz.	FWHM	D_{hkl}
1 1 0	5497,116	26,5772	1	0,854	10
1 0 1	4140,79	33,8872	0,8898	0.915	9
2 0 0	1601,142	38,0082	1	0.955	9
2 1 1	4252,853	51,8407	1	1.12	8
2 2 0	920,3923	54,6573	0,7964	1.205	7
3 1 0	1383,989	62,0447	1	1.319	7
1 1 2	676,6418	64,7226	0,376	1.311	7
3 0 1	1410,859	65,9959	1	1.271	7
2 0 2	406,4227	71,4813	0,7425	1.255	7
3 2 1	817,0611	78,8599	0,6645	1.599	6

Table S8. Results of individual profile fitting of the cassiterite phase in the SN-0 sample (program XFIT), and the corresponding D_{hkl} values calculated from the Scherrer equation.

hkl	Area	2 Θ	Lortz.	FWHM	D_{hkl}
1 1 0	6350,169	26,5601	0,8856	0.276	30
1 0 1	5057,013	33,8601	0,8542	0.284	29
2 0 0	1379,944	37,947	0,7614	0.281	30
1 1 1	189,4007	38,9854	0,0039	0.287	29
2 1 1	4564,181	51,785	0,8783	0.298	30
2 2 0	1038,563	54,7663	0,7523	0.306	29
0 0 2	531,5119	57,8605	1	0,267	34
3 1 0	1019,263	61,8989	0,9322	0,278	30
1 1 2	1107,751	64,7677	1	0,269	34
3 0 1	1354,293	65,9924	0,8207	0,323	29
2 0 2	549,4526	71,3059	1	0,287	34
3 2 1	1030,068	78,744	1	0,339	29

Table S9. Results of Williamson-Hall analysis of the hematite phase in samples FE-0, FE-1, FE-2 and FE-3.

SAMPLE	D_v^0 / nm	*e*

FE-0	26	$1×10^{-4}$
FE-1	28	$1×10^{-4}$
FE-2	21	$2×10^{-4}$
FE-3	23	$4×10^{-4}$

Table S10. Results of Williamson-Hall analysis of cassiterite phase in samples SN-0, SN-1 and SN-2.

SAMPLE	D_v^0 / nm	e
SN-0	29*	$<1×10^{-4}$
SN-1	28	$1×10^{-4}$
SN-2	13	$4{,}2×10^{-3}$

* Williamson-Hall analysis of the cassiterite phase in the SN-0 sample indicated the presence of size anisotropy wherein the diffraction lines with the Miller indices *hk*2 are somewhat narrower (D_v value in the direction of *hk*2 was estimated at 34 nm).

3. STEM images with particle size distributions and HAADF images of selected samples

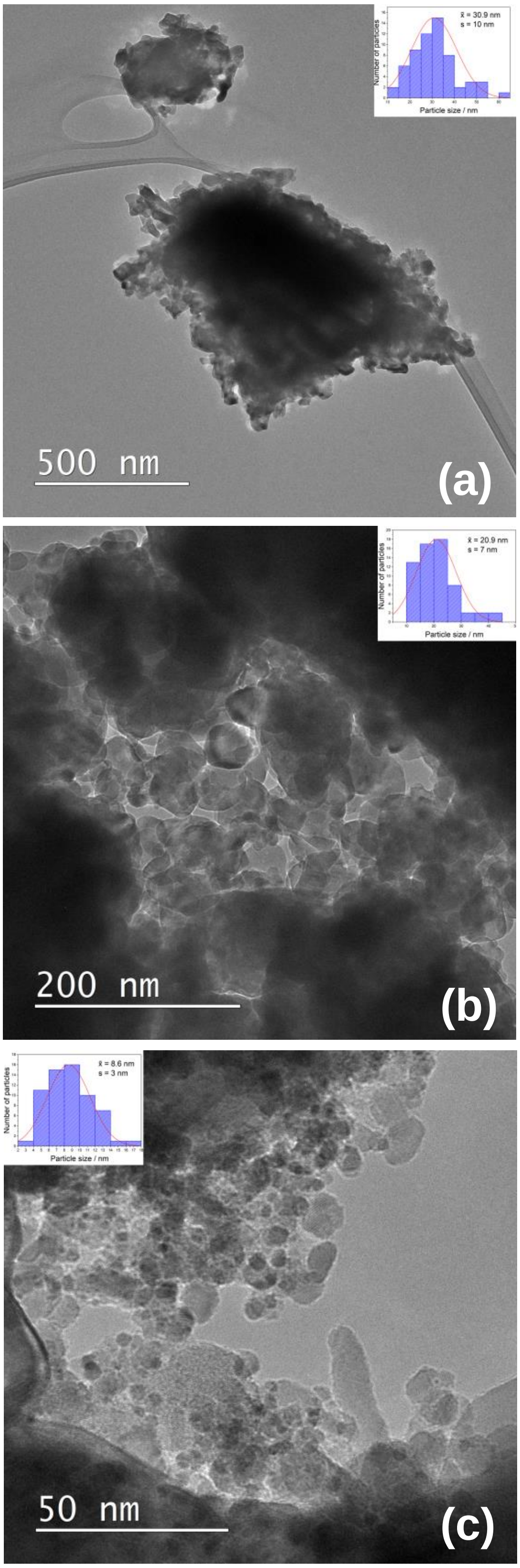

Figure S6. STEM images with particle size distributions (inset) of samples FE-1 (**a**), FE-2 (**b**) and FE-3 (**c**). The mean particle sizes of 30.9, 20.9 and 8.6 nm were measured for samples FE-1, FE-2 and FE-3, respectively.

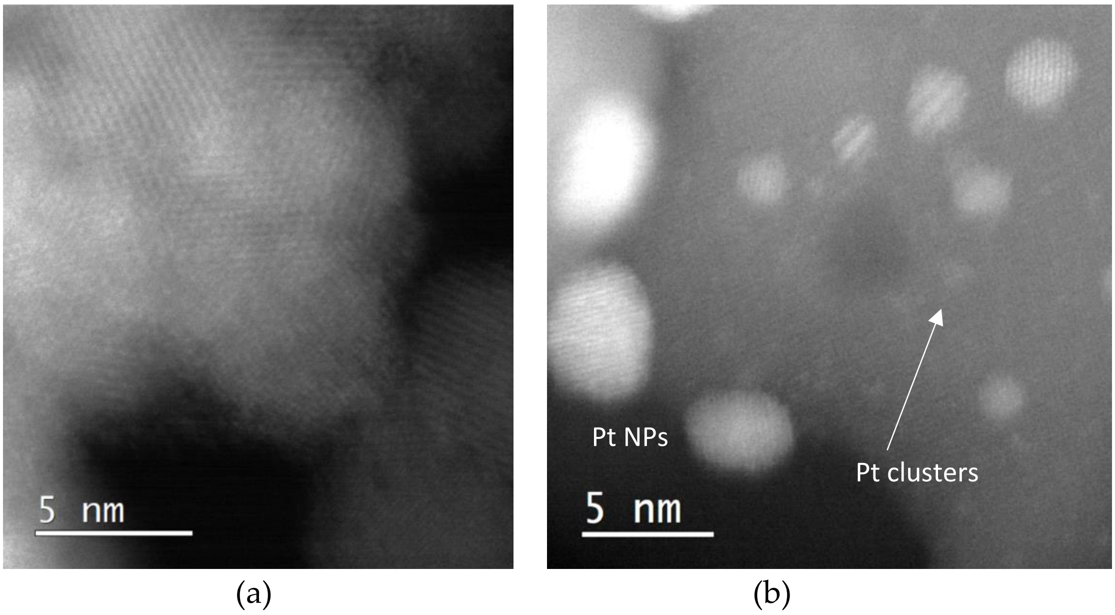

Figure S7. HAADF images of samples SN-1 (a) and sample FE-1 (b). HAADF image of sample SN-1 shows uniform contrast, whereas in sample FE-1 Pt nanoparticles and cluster are clearly visible. Sn and Pt have very different atomic numbers (50 vs. 78) and the intensity of the HAADF image (besides the thickness) is approximately related to $Z^{1.7}$. In the case of Pt-based particles or Pt-rich surface layers, this should be seen as areas of higher contrast. At the same time, the EDXS analysis shows 2-3 wt% Pt in these areas (so these areas are not pure SnO2).

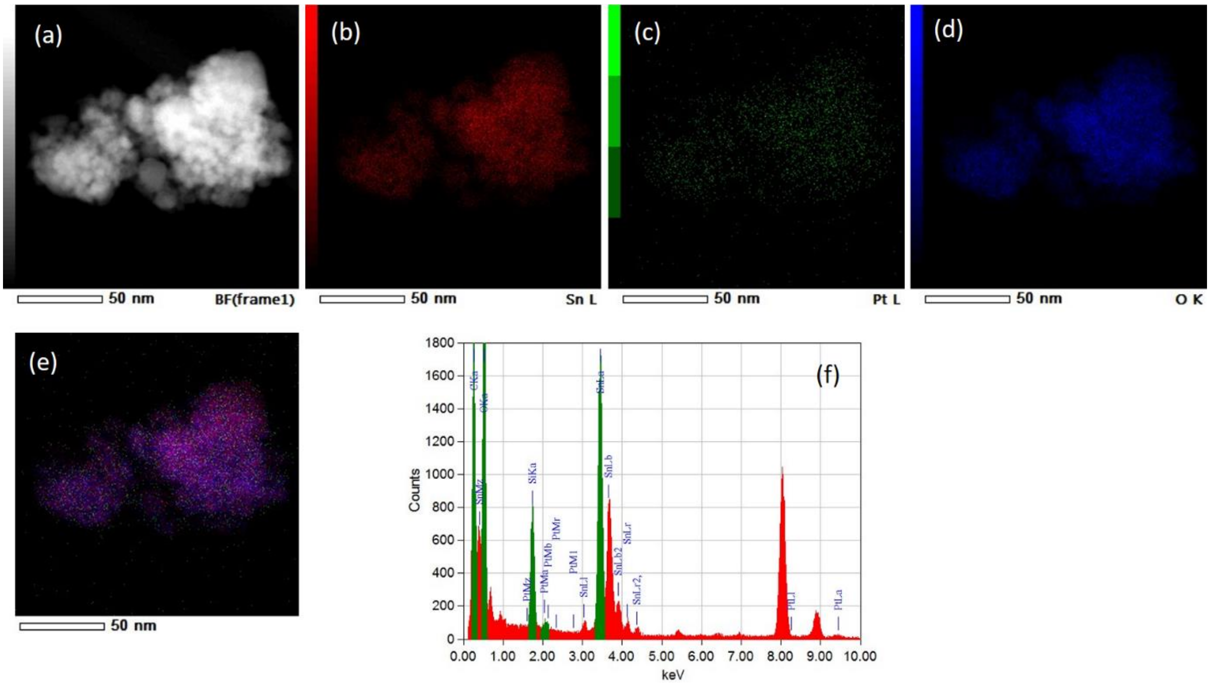

Figure S8. STEM image of sample SN-2 (**a**) and corresponding EDXS elemental mapping images of Sn L edge (**b**), Pt M edge (**c**), O K edge (**d**) and overlay of Sn L, Pt M and O K edges (**e**). EDXS spectrum of sample SN-1 (**f**). It can be seen that all three elements are homogeneously dispersed and that there are no distinct Pt clusters.

4. References

[1] G. Štefanić, S. Krehula, I. Štefanić, Chem. Commun. 49 (2013) 9245–9247.

[2] R. W. Cheary, A. A. Coelho, J Appl. Cryst. 25 (1992) 109-121.

[3] G.K. Williamson, W.H. Hall, Acta Metallurgica. 1 (1953) 22–31.
